# Exposure to sub-chronic and long-term particulate air pollution and heart rate variability in an elderly cohort: the Normative Aging Study

**DOI:** 10.1186/s12940-015-0074-z

**Published:** 2015-11-06

**Authors:** Irina Mordukhovich, Brent Coull, Itai Kloog, Petros Koutrakis, Pantel Vokonas, Joel Schwartz

**Affiliations:** Exposure, Epidemiology and Risk Program, Department of Environmental Health, Harvard School of Public Health, Landmark Center, 401 Park Dr, Boston, MA 02215 USA; Department of Biostatistics, Harvard School of Public Health, Boston, MA USA; Department of Geography and Environmental Development, Ben-Gurion University of the Negev, Beer Sheva, Israel; VA Normative Aging Study, Veterans Affairs Boston Healthcare System and the Department of Medicine Boston University School of Medicine, Boston, MA USA; Exposure, Epidemiology and Risk Program, Department of Environmental Health, Harvard School of Public Health, and Channing Laboratory, Brigham and Women’s Hospital, Harvard Medical School, Boston, MA USA

**Keywords:** Epidemiology, Heart rate variability, Air pollution, Particulate matter

## Abstract

**Background:**

Short-term particulate air pollution exposure is associated with reduced heart rate variability (HRV), a risk factor for cardiovascular morbidity and mortality, in many studies. Associations with sub-chronic or long-term exposures, however, have been sparsely investigated. We evaluated the effect of fine particulate matter (PM_2.5_) and black carbon (BC) exposures on HRV in an elderly cohort: the Normative Aging Study.

**Methods:**

We measured power in high frequency (HF) and low frequency (LF), standard deviation of normal-to-normal intervals (SDNN), and the LF:HF ratio among participants from the Greater Boston area. Residential BC exposures for 540 men (1161 study visits, 2000–2011) were estimated using a spatio-temporal land use regression model, and residential PM_2.5_ exposures for 475 men (992 visits, 2003–2011) were modeled using a hybrid satellite based and land-use model. We evaluated associations between moving averages of sub-chronic (3–84 day) and long-term (1 year) pollutant exposure estimates and HRV parameters using linear mixed models.

**Results:**

One-standard deviation increases in sub-chronic, but not long-term, BC were associated with reduced HF, LF, and SDNN and an increased LF:HF ratio (e.g., 28 day BC: −2.3 % HF [95 % CI:−4.6, −0.02]). Sub-chronic and long-term PM_2.5_ showed evidence of relations to an increased LF and LF:HF ratio (e.g., 1 year PM: 21.0 % LF:HF [8.6, 34.8]), but not to HF or SDNN, though the effect estimates were very imprecise and mostly spanned the null.

**Conclusions:**

We observed some evidence of a relation between longer-term BC and PM_2.5_ exposures and changes in HRV in an elderly cohort. While previous studies focused on short-term air pollution exposures, our results suggest that longer-term exposures may influence cardiac autonomic function.

**Electronic supplementary material:**

The online version of this article (doi:10.1186/s12940-015-0074-z) contains supplementary material, which is available to authorized users.

## Background

Particulate air pollution exposure is associated with cardiovascular mortality and morbidity in a multitude of epidemiologic studies, including at levels at or below EPA standards [1, 2]. Pathways potentially mediating this association include induction of systemic inflammation [3, 4] and oxidative stress [5, 6], as well as changes in ion channel function in myocardial cells [7] or cardiac autonomic function [8–10].

Heart rate variability (HRV) is a marker of cardiac autonomic control, which reflects autonomic modulation of the rhythmic activity of the sinus node [11]. Reduced HRV is predictive of increased cardiovascular morbidity and mortality risk [12, 13]. Short-term particulate air pollution exposure is associated with reduced HRV in many epidemiologic studies, including a recent meta-analysis [14] and within the current study population [10, 15]. Associations are particularly pronounced among the elderly [16], among those with preexisting cardiovascular disease or diabetes [17], or among people with reduced antioxidative defenses [17, 18]. However, the association between exposure to sub-chronic or chronic air pollution exposure and HRV is unclear, although long-term exposure is known to have a stronger effect on cardiovascular mortality than acute exposure [19]. Also, many previous studies have used air pollution at a central site as the exposure metric, introducing potential exposure error.

Particulate matter is a complex mixture of particles and liquids, traffic and non-traffic components, and varies in composition across geographic regions [1]. Studies to date suggest that traffic-related particulate pollution may contribute significantly to cardiovascular outcomes [17, 20], including changes in HRV [19].

In the current study, we used address-specific estimates of exposure and evaluated whether sub-chronic (3–84 day) and long-term (1 year) exposure to ambient particulate matter <2.5 μm in aerodynamic diameter (PM_2.5_) or black carbon (BC), a marker for traffic related air pollution, would be associated with reduced HRV in a cohort of elderly men. In addition to examining the main effect of particulate air pollution on HRV, we also evaluated potential modification by oxidative stress allelic profile, hypertension, obesity and diabetes.

## Methods

### Study population

Our analysis included 540 men enrolled in the Veterans Administration Normative Aging Study (NAS) who had complete information regarding ambient BC concentrations, HRV measures, and all covariates of interest, and who underwent 1161 study visits between November 14, 2000 and August, 24, 2011. We also evaluated a largely overlapping group of 475 participants with complete information regarding PM_2.5_ concentrations and all covariates, who presented for 992 study visits between January 14, 2003 and December 21, 2011. The NAS is a prospective cohort study, described in detail previously [21]. Briefly, this closed cohort was established in 1963 and enrolled 2280 adult male volunteers, free of chronic medical conditions, who were living in the Greater Boston area. Detailed questionnaires and physical examinations were administered at all center-based study visits, occurring every 3–5 years. Loss to follow-up has been predominantly due to death or moving out of the study area.

There were 727 active NAS study participants during the time period of interest. Of these, 596 participants had complete information regarding sub-chronic or long-term residential BC exposures and HRV measures for one or more study visits, and 540 had complete information regarding PM_2.5_ exposures and HRV measurements. We excluded study participants with problematic heart rate measurements from the analysis (as described below, *n* = 56 for BC and *n* = 65 for PM_2.5_), which brought the final sample size to 540 for BC and 475 for PM_2.5_. Participants presented for 1–4 visits, with 65.6 % (*n* = 354) undergoing at least two study visits for BC and 65.1 % (*n* = 309) for PM_2.5_.

Physical examinations included measurement of height and weight, which was used to calculate body mass index (BMI, in kg/m^2^). Blood samples were collected to assess fasting blood glucose (FBG) levels. Smoking history was obtained via an American Thoracic Society questionnaire. Participants’ diabetes status was assessed based on a physician’s diagnosis of type 2 diabetes, and/or use or diabetes medication assessed during the physician interview. Similarly, hypertension was defined as a measured systolic blood pressure (SBP) of ≥140 mmHg, a measured diastolic blood pressure (DBP) of ≥90 mm Hg, or participant use of anti-hypertensive medication. SBP and DBP were measured by a physician, and mean arterial blood pressure (MAP) was defined as DBP + 1/3(SBP-DBP). Finally, room temperature was recorded at the time of electrocardiogram (ECG) measurement.

This study was approved by the Harvard School of Public Health and Veteran Administration institutional review boards, and all participants provided their written informed consent.

To summarize, all participants were administered questionnaires and were examined by a physician at each study visit. These examinations included measurement of heart rate variability, described below. Outdoor temperature and residential air pollution exposure estimates were also evaluated for several time periods preceding a participants’ study visit and are described below.

### Heart rate variability

HRV was measured between 6:00 AM and 1:00 PM using a two-channel, five-lead ECG monitor (Trillium 30,000; Forest Medical, East Syracuse, NY), as described in detail previously [10, 22]. Briefly, participants rested for 5 min, and then remained seated during the ECG, which was recorded at a sampling rate of 256 Hz per channel for approximately 7 min. Heart rate and HRV measures were then calculated using PC-based software (Trillium 3000 PC Companion Software for MS Windows; Forest Medical, East Syracuse, NY) and reviewed by an experienced scanner to correct for any errors. The following HRV measures were computed: high-frequency power (HF; 0.15–0.4 Hz), low-frequency power (LF; 0.04–0.15 Hz), LF:HF ratio, and the standard deviation of normal-to-normal intervals (SDNN). HF is a marker of parasympathetic drive, LF is a marker of both sympathetic and para-sympathetic activity, and the LF:HF ratio reflects the balance of sympathetic and para-sympathetic systems and may also be influenced by baroreflexes. In contrast to the latter frequency domain measures, SDNN is a time domain measure that reflects the variability in N-N interval duration. As mentioned above, participants with problematic heart rate measurements (i.e., atrial fibrillation, atrial bigeminy/trigeminy, pacemakers, irregular rhythm, irregular sinus rhythm, frequent ventricular ectopic activity, ventricular bigeminy, multifocal atrial tachycardia, or a measurement time of less than 3.5 min) were excluded from our analysis.

### Air pollution and weather

Daily BC exposures at participants’ residences in the greater Boston area was estimated using a validated spatio-temporal land-use regression model [23]. Daily BC predictions were obtained using estimates averaged from 148 monitoring sites, local meteorological conditions, land use variables (including traffic density), day of the week, and other factors [24]. Spatially-resolved daily residential PM_2.5_ exposures were also estimated at each residence using a validated hybrid exposure model, which incorporated satellite based aerosol optical depth (AOD) measurements as well as land-use and meteorological variables (i.e., outdoor temperature, elevation, visibility, wind speed, distance to major roads, percent of open space, traffic density, proximity to point emissions, and area emissions). More in depth details can be found in Kloog et al. [25].

For this study, we evaluated moving averages of pollutant exposure at 3, 7, 21, 14, 28, 56 and 84 days as well as 1 year prior to each study visit. Specifically, we calculated each participant’s average pollutant exposure level during a given number of a days before each study visit. We also calculated BC and PM_2.5_ levels 1 and 2 days prior to the study visits, for use in sensitivity analyses.

Our regression models accounted for both room temperature and outdoor temperature. Apparent temperature is defined as a person’s perceived outdoor temperature in °C ([26, 27], and calculated using the following formula: −2.653 + (0.994 * air temperature) + (0.0153 * dew-point temperature)). We calculated moving averages of apparent temperature, corresponding to 1–28 days before each study visit. Measured outdoor temperature was also averaged during the two months, three months, and one year preceding each study visit.

### Oxidative stress genetic scores

Oxidative stress allelic profiles were calculated using a genetic score approach [28], in which genetic variants were selected using the least absolute shrinkage and selection operator (Lasso) based on their relation to 8-hydroxydeoxyguanosine levels (8-OhdG, a marker of oxidative DNA damage) [28]. The genetic variants used to calculate oxidative stress score were *CAT* (rs1001179, rs480575), *GC* (rs2282679), *GCLM* (rs3170633)*, HMOX1* (rs2071746, rs5995098), and *NQO1* (rs1800566). Scores representing participants’ allelic profiles were constructed by summing these genetic variants, using the signs of the coefficients of the Lasso penalization [28].

### Statistical analyses

We evaluated the association between one standard deviation (SD) increases in moving averages of BC and PM_2.5_ exposure estimates (3 days-1 year) and several HRV measures (HF, LF, LF:HF, and SDNN), using linear mixed models with random intercepts and a compound-symmetry covariance structure in a repeated measures study. The outcome of interest was percent change in HRV, which was calculated as [10^(β*SD)-^1]*100 %; 95 % confidence intervals (CIs) were calculated as [10^[SD*(β±1.96*SE)]^-1]*100 %, where SE is the standard error associated with the regression coefficient β [10]. Measures of HRV were log-transformed to improve normality and stabilize the variance.

All models controlled for potential confounders, which were identified following a thorough literature search: age, BMI, FBG, smoking history (current, former, or never), current use of anti-hypertensive medications (yes/no), room temperature at the time of ECG measurement, season (indicated using the sine and cosine of the date), MAP, and moving averages of outdoor temperature corresponding to the pollutant exposure measurement interval of interest (using both a linear and quadratic term).

Because of previous research indicating potentially susceptible subgroups [10, 15], we examined effect modification of the association between 28 day exposure to BC and HRV parameters by obesity (defined as BMI ≥ 30 kg/m^2^; yes/no), hypertension (yes/no), diabetes (yes/no) and oxidative stress allelic profile (high/low; dichotomized based on median score) using separated linear regression models. We also included multiplicative interaction terms in regression models to evaluate effect modification. We conducted all analyses using SAS versions 9.3 and 9.4.

#### Survivor bias

Because people who did not return for subsequent visits are likely to have been less healthy than those who did, this study, like all longitudinal cohorts, can suffer from a dynamic selection bias. To counteract this, we used inverse probability weighting. Specifically, we modeled the probability of returning for a visit based on variables available at previous visits, including HRV measures. The observations were given weights of one for the first visit, the inverse of the probability of returning for the second visit, and the product of the inverse of the probability of returning for the second visit times the inverse of the probability of returning for the third visit, and so on for subsequent visits.

## Results

Participants’ characteristics at baseline visit are reported in Table 1. Briefly, at this initial examination, participants evaluated for the association between BC and HRV presented with a mean age of 73.6 years; most (71 %) were former or current smokers, 20 % were diabetic and 25 % had a BMI > 30 kg/m^2^. Participants evaluated for the association between PM_2.5_ and HRV presented with a mean age of 75.2 years; most (70 %) were former or current smokers, 21 % were diabetic and 26 % had a BMI > 30 kg/m^2^. We also present participants’ baseline HRV measures (HF, LF, SDNN, and the LF:HF ratio) in Table 1.

**Table 1:** Characteristics at baseline of study participants with information on BC (*n* = 540) and PM_2.5_ (*n* = 475)^a^

Study variable	Subjects with BC^b^	Subjects with PM_2.5_^b^
Age (years)	73.6 (7.0)	75.2 (6.4)
Body mass index (kg/m^2^)	28.1 (4.0)	27.9 (4.1)
≥ 30 kg/m^2^ (n [%])	137 (25.4)	121 (25.5)
< 30 kg/m^2^ (n [%])	403 (74.6)	354 (74.5)
Fasting blood glucose (n [%])		
< 110 mg/dL	371 (68.7)	332 (69.9)
≥ 110 or <126 mg/dL	96 (17.8)	91 (19.2)
≥ 126 mg/dL	73 (13.5)	52 (11.0)
Smoking		
Never smoker	156 (28.9)	143 (30.1)
Current smoker	22 (4.1)	14 (3.0)
Former smoker	362 (67.0)	318 (67.0)
Anti-hypertensive medicine use (n [%])		
Yes	325 (60.2)	311 (65.5)
No	215 (39.8)	164 (34.5)
Season		
Spring	135 (25.0)	112 (23.6)
Summer	109 (20.2)	120 (25.3)
Fall	166 (30.7)	144 (30.3)
Winter	130 (24.1)	99 (20.8)
Mean arterial pressure (mmHg)	92.6 (10.7)	87.9 (10.0)
Room temperature (°C)	24.1 (1.9)	23.3 (1.8)
Diabetes (n [%])		
Yes	107 (19.8)	99 (20.8)
No	433 (80.2)	376 (79.2)
Oxidative stress gene score		
Low	184 (42.0)	146 (38.4)
High	254 (58.0)	234 (61.6)
Heart rate variability		
Log_10_HF, msec^2^	1.9 (0.7)	2.0 (0.7)
Log_10_LF, msec^2^	2.0 (0.6)	1.9 (0.6)
Log_10_SDNN, msec	1.5 (0.3)	1.5 (0.3)
Log_10_LF:HF	0.04 (0.5)	−0.05 (0.5)

*BC* black carbon, *PM*_*2.5*_ particulate matter <2.5 μm in aerodynamic diameter

^a^Normative Aging Study (2000–2011)

^b^Values are mean (SD) or n (%)

Pollutant concentrations across all study visits and averaging periods are reported in Table 2. For example, for a 28 day moving average of BC exposure, we observed a mean exposure level of 0.4 μg/m^3^ (SD: 0.2; 5^th^–95^th^ percentiles: 0.2–0.8 μg/m^3^); for PM_2.5_, the corresponding values were 9.5 μg/m^3^ (SD: 2.5; 5^th^–95^th^ percentiles: 5.8–14.2 μg/m^3^). For a 3 day moving average of BC exposure, we observed a mean exposure level of 0.4 μg/m^3^, with a standard deviation of 0.3. We report Spearman correlation coefficients between selected exposure measure durations (3, 14, 28 and 84 days, as well as 1 year) for both BC and PM_2.5_ in Additional file 1: Table S1. For BC, correlations between exposure measurement periods were fairly high, ranging from 0.65 to 0.97. For PM_2.5_, correlations were somewhat weaker, ranging from 0.25 to 0.87. The correlation between 28 day BC exposure and 1 year BC exposure was *r* = 0.76, and the corresponding correlation for PM_2.5_ was *r* = 0.53.

**Table 2:** BC and PM_2.5_ exposure distributions for Normative Aging Study participants^a^

Pollutants (moving averages)	N visits	Mean (SD)	5^th–^95^th^ percentiles	IQR
BC (μg/m^3^)				
3 days	1161	0.43 (0.29)	0.12–0.93	0.29
7 days	1161	0.44 (0.26)	0.14–0.85	0.28
14 days	1160	0.44 (0.25)	0.14–0.81	0.26
21 days	1153	0.44 (0.24)	0.14–0.81	0.26
28 days	1148	0.44 (0.24)	0.15–0.81	0.26
1 year	974	0.45 (0.17)	0.22–0.74	0.19
PM_2.5_ (μg/m^3^)				
3 days	920	9.40 (4.48)	3.79–18.60	5.44
7 days	918	9.41 (3.32)	4.74–15.94	4.44
14 days	920	9.46 (2.91)	5.30–14.61	3.95
21 days	916	9.51 (2.67)	5.62–14.50	3.59
28 days	911	9.49 (2.53)	5.82–14.15	3.55
1 year	806	9.71 (1.36)	7.40–11.64	2.01

*BC* black carbon, *IQR* interquartile range, *PM*_*2.5*_ particulate matter <2.5 μm in aerodynamic diameter, *SD* standard deviation

^a^Study visits with information on BC spanned 2000 and 2011. Study visits with information on PM_2.5_ spanned 2003 and 2011

Associations between moving averages of BC and PM_2.5_ exposures and HRV parameters are presented in Table 3. We observed associations between increased sub-chronic BC exposure and decreased HF, LF, and SDNN, as well as an increased LF:HF ratio, though the confidence intervals were somewhat imprecise and in most cases spanned the null. This association was slightly stronger when evaluating 28-day and 56-day moving averages of exposure relative to other exposure durations, and was strongest for HF in comparison to other measures of HRV. For example, a 1 SD increase in BC measured during the preceding 28 days was associated with a 2.3 % decrease in HF (95 % CI: −4.6, −0.02), a 1 SD increase in 21 day BC exposure was associated with a 2.2 % decrease in HF (95 % CI: −4.4, 0.1), and a 1 SD increase in 7 day BC exposure was associated with a 1.9 % decrease in HF (95 % CI: −4.3, 0.6). A 1 SD increase in 28 day BC exposure was also associated with a 1.0 % decrease in LF (95 % CI: −3.0, 1.0), a 0.5 % decrease in SDNN (95 % CI: −1.5, 0.4), and a 1.3 % increase in the LF:HF ratio (95 % CI: −0.2, 2.8). When evaluating long-term BC exposures of 1 year prior to study visit, results showed a similar pattern but were attenuated greatly (Table 3). Moreover, when we included 28 day and 1 year BC exposure in the same model for HF, the magnitude of the association with 28 day BC increased, while the association with 1 year BC changed direction (data not shown). Similarly, effect estimates for 28 day BC were strengthened when adjusting for BC exposure 1 day before the visit (data not shown), indicating that short-term exposures are not dominating the observed association with sub-chronic BC.

**Table 3:** Mixed linear effects models estimating percent changes in HRV measures according to one standard deviation increases in BC and PM2.5 levels^a,b^

Exposure duration	HRV parameter	BC		PM_2.5_	
		% change	95 % CI	% change	95 % CI
3 days	HF	−2.1	−4.8, 0.6	−35.5	−63.0, 12.5
	LF	−1.0	−3.3, 1.4	−31.7	−57.5, 9.5
	SDNN	−0.4	−1.5, 0.8	−11.5	−29.6, 11.4
	Ratio (LF:HF)	1.1	−0.6, 2.9	6.5	−25.4, 52.0
7 days	HF	−1.9	−4.3, 0.6	4.3	−31.2, 58.2
	LF	−0.7	−2.8, 1.5	9.4	−23.4, 56.1
	SDNN	−0.4	−1.4, 0.6	3.1	−13.2, 22.4
	Ratio (LF:HF)	1.1	−0.5, 2.7	4.4	20.1, 36.4
14 days	HF	−2.2	−4.5, 0.3	1.1	−30.5, 47.1
	LF	−0.9	−2.9, 1.2	15.6	−16.2, 59.4
	SDNN	−0.4	−1.4, 0.6	6.6	−8.7, 24.5
	Ratio (LF:HF)	1.3	−0.3, 2.9	13.1	−10.8, 43.5
21 days	HF	−2.2	−4.4, 0.1	−6.8	−34.5, 32.6
	LF	−1.2	−3.2, 0.9	15.0	−15.0, 55.6
	SDNN	−0.6	−1.6, 0.4	6.8	−7.6, 23.5
	Ratio (LF:HF)	1.1	−0.5, 2.7	21.6	−2.7, 51.9
28 days	HF	−2.3	−4.6, −0.02	−8.2	−34.8, 29.2
	LF	−1.0	−3.0, 1.0	22.4	−8.6, 64.1
	SDNN	−0.5	−1.5, 0.4	7.8	−6.3, 24.0
	Ratio (LF:HF)	1.3	−0.2, 2.8	31.4	5.9, 63.0
56 days	HF	−2.3	−4.6, −0.1	−13.1	−34.6, 15.4
	LF	−1.0	−3.0, 1.0	11.2	−13.0, 42.0
	SDNN	−0.7	−1.6, 0.3	2.5	−8.9, 15.4
	Ratio (LF:HF)	1.3	−0.2, 2.8	25.0	4.5, 49.6
84 days	HF	−2.0	−4.2, 0.2	−2.6	−24.3, 25.4
	LF	−0.9	−2.8, 1.1	16.1	−6.6, 44.1
	SDNN	−0.7	−1.6, 0.3	6.0	−4.5, 17.7
	Ratio (LF:HF)	1.2	−0.3, 2.6	16.2	−1.0, 36.5
1 year	HF	−0.3	−2.1, 1.6	1.7	−14.6, 21.2
	LF	−0.2	−1.7, 1.4	23.6	6.0, 44.1
	SDNN	−0.1	−0.9, 0.7	7.0	−0.5, 15.1
	Ratio (LF:HF)	0.04	−1.1, 1.2	21.0	8.6, 34.8

*BC* black carbon, *HF* high frequency, *LF* low frequency, *PM*_*2.5*_ particulate matter <2.5 μm in diameter, *SD* standard deviation, *SDNN* standard deviation of normal-to-normal intervals

^a^Specifically, we evaluated associations between percent change in HRV and 1 SD increases in BC (1161 visits) and PM_2.5_ (992 visits)

^b^Study visits with information on BC spanned 2000 and 2011. Study visits with information on PM_2.5_ spanned 2003 and 2011

We found some evidence of associations between increased short-term PM_2.5_ exposure estimates and decreases in HF, LF, and SDNN, as well as increased LF:HF ratio (our results for a 3 day moving average are reported in Table 3; data for 1 and 2 day averages are not shown), though the associated confidence intervals were very wide and many of the effect estimates were not statistically significant. In addition, the patterns of association we observed for shorter-term exposures were inconsistent when evaluating associations between modeled PM_2.5_ exposures reflecting 7 day to 1 year moving averages and HF and SDNN (Table 3). Associations between sub-chronic and long-term PM_2.5_ and both LF and the LF:HF ratio were consistently elevated and tended to be strongest for longer-term exposures. For example, the percent increase in the LF:HF ratio in relation to a 1 SD increase in PM_2.5_ was 13.1 % (95 % CI: −10.8, 43.5) for a 14 day moving average, 31.4 % (95 % CI: 5.9, 63.0) for a 28 day moving average, and 21.0 % (95 % CI: 8.6, 34.8) for a 1 year average. The corresponding increases for LF were 15.6 % (95 % CI: −16.2, 59.4), 22.4 % (95 % CI: −8.6, 64.1), and 23.6 % (95 % CI: 6.0, 44.1, respectively (Table 3).

Finally, associations between 28 day BC exposure estimates and HF stratified by obesity, hypertension, diabetes, and oxidative stress genetic score are reported in Table 4. We also examined interactions between 28 day BC and the aforementioned characteristics with respect to other HRV measures (data not shown). No multiplicative interaction terms rose to the level of statistical significance and confidence intervals were wide. Consistent with previous findings, however, the decrease in HRV in relation to BC exposure was greater among those with obesity or diabetes. For example, a 1 SD increase in BC exposure was associated with a 1.7 % decrease in HF (95 % CI: −4.5, 1.2) among men with BMI <30, and a 3.5 % decrease in HF among men with a BMI of 30 or greater (95 % CI: −7.4, 0.5) (Table 4). BC exposure was also associated with a greater increase in HF among those with a lower oxidative stress genetic score. We observed no consistent patterns for hypertension.

**Table 4:** Modification of the association between a one standard deviation increase in 28-day BC and percent change in HF^a^

Characteristics	% change	95 % CI
Oxidative stress genetic score < median	−3.3	−7.4, 1.0
Oxidative stress genetic score > median	−1.8	−4.9, 1.5
P for interaction	0.55	
Not obese	−1.7	−4.5, 1.2
Obese	−3.5	−7.4, 0.5
P for interaction	0.64	
No diabetes	−2.1	−4.6, 0.4
Diabetes	−4.6	−10.6, 1.8
P for interaction	0.51	
No hypertension	−2.2	−5.8, 1.5
Hypertension	−2.2	−5.0, 0.8
P for interaction	0.60	

*BC* black carbon, *HF* high frequency power, *SD* standard deviation

^a^We evaluated effect modification of the association between a 1 SD increase in the 28 day moving average of BC exposure and percent change in HF; Normative Aging Study, 2000–2011

## Discussion

We report positive associations between several measures of sub-chronic BC exposure, a marker of traffic pollution, and decreased HRV in a cohort of elderly men, although confidence intervals were wide and mostly spanned the null. These findings are consistent with many previous studies reporting associations between short-term particulate pollution exposure and decreased HRV [10, 14], which is predictive of increased cardiovascular morbidity and mortality [12, 13], but extend the literature to longer exposure measurement periods, and demonstrate somewhat larger effect sizes for those longer exposures. Longer-term exposures may be of most interest when assessing risk for adverse cardiovascular outcomes [19]. It is also notable that the observed associations reflect not only short-term spikes in particulate levels, but rather occur at particulate pollutant levels that are consistently at or below EPA standards. Our also extends the literature by using a sophisticated modeling approach, rather than fixed monitoring data, to predict individualized residential pollutant exposures during 3, 7, 14, 21, 28, 56 and 84 days and well as 1 year prior to each study visit [23, 25]. When evaluating BC exposures, we consistently found a larger effect size for HF, a marker of parasympathetic drive, than for LF. We also found some evidence of an increased LF:HF ratio. Recent research suggests the LF:HF ratio may be influenced primarily by parasympathetic output and baroreflex function [29, 30].

We observed attenuated associations between HF and 1-year BC exposure, which changed direction upon adjustment for 28-day BC exposure, suggesting the weak association observed between 1 year BC and HRV was explained by correlations with the shorter-term, sub-chronic exposures rather than by the long-term exposure. We did not observe a consistent pattern of associations between sub-chronic or long-term PM_2.5_ exposure and HF. While studies consistently report associations between short-term particulate pollution exposure and reduced HF, especially among the elderly or those with preexisting conditions [14, 16], very few have examined exposure periods of greater than a few days [31, 32]. Hence, although long-term PM exposure is known to have a stronger effect on cardiovascular mortality than acute exposure [19], the effects of long-term or sub-chronic particulate exposure on HF are unclear. Specifically, to our knowledge, only two studies have examined the association between long-term PM exposure (specifically, PM <10 μm in aerodynamic diameter [PM_10_] levels averaged over a 10 year period, among participants aged 50 and over) and HRV; they reported a null overall association between long-term PM_10_ and HRV, with reduced HRV observed among participants taking ACE inhibitors or with a particular pro-inflammatory polymorphism [31, 32]. Ours is also the first study to evaluate sub-chronic PM_2.5_ or BC exposure in relation to HRV. Further studies are necessary to clarify the effect of longer-term pollutant exposure on cardiac autonomic control.

We report discrepant findings for BC and PM_2.5_. The direction of results for modeled PM_2.5_ exposure 1, 2, or 3 days prior to the study visit are consistent with previous fixed monitoring studies from the NAS [10, 15] as well as with our findings for sub-chronic BC exposure. Air pollution was associated with decreased HF, LF, and SDNN as well as an increased LF:HF ratio in these studies. In our study, associations between longer-term PM_2.5_ levels and HF measures were inconsistent, and PM_2.5_ was related to an increase in LF, which is inconsistent with previous studies among elderly people, but is sometimes seen among younger subjects [33, 34]. We also observed a consistent, though imprecise, positive association between PM_2.5_ exposure and an increased LF:HF ratio, which was similar to our results for BC and was in general strongest for exposures measured over longer time periods.

Although BC is a component of PM, the source profiles of BC and PM_2.5_ differ. BC is a marker of traffic pollution, especially diesel exhaust and, in Boston, home heating oil [35], whereas these sources contribute to a minority of PM_2.5_ levels [36, 37]. The effects of different particle components or sources on health outcomes may vary, and emerging evidence suggests that traffic pollution may be especially important with regard to cardiovascular disease [38]. It should be noted that BC and PM_2.5_ estimates were derived from different exposure models; the differences in results for BC and PM_2.5_ and for shorter and longer-term PM_2.5_ do not have a clear biological basis and could also be due to a difference in modeling approach. We also note that that confidence intervals for the PM_2.5_ estimates were very wide, which increases the likelihood some of our results may be due to chance. While we cannot be sure of an underlying mechanism, one possible reason that we observed stronger associations for sub-chronic rather than long-term BC could be that HRV fluctuates based on a variety of short-term stimuli and this might overpower the effect of air pollution exposure over the course of a year, but not a shorter period of 1–2 months.

Potential mechanisms underlying possible associations between BC or PM_2.5_ and HRV include a particulate-induced increase in pulmonary oxidative stress, which can in turn induce proinflammatory cytokines [39], increase extracellular calcium influx [40], inactivate nitric oxide [41], and lead to greater parasympathetic than sympathetic nervous system withdrawal [19]. The result of these changes is an increased risk of cardiovascular morbidity and mortality, including ventricular arrhythmias and myocardial infarction [19].

We did not observe strong evidence of effect modification by obesity, hypertension, diabetes, or oxidative stress allelic profile, which may have been due to the low precision of stratified effect estimates. While not rising to the level of statistical significance, associations between BC exposure and HRV were somewhat stronger among men with obesity and diabetes, which is notable mainly because of similarity to previous findings [10, 15]. One explanation for these findings is that diabetes and obesity are themselves associated with reduced autonomic function [42–44], possibly rendering diabetics and those with a BMI over 30 kg/m^2^ more susceptible to the effects of particles on this same endpoint. We observed a stronger reduction in HF relative to BC exposure among men with lower oxidative stress genetic scores, which was unexpected. It should be noted that we did not have sufficient power to examine effect modification in an optimal way, and larger studies will be needed in the future in order to examine the possibility of susceptible subgroups more thoroughly, and to obtain more precise and stable effect estimates than we were able to calculate.

We acknowledge several limitations of our study. These include potential exposure misclassification of pollutant estimates, which could affect precision and potential bias effect size [45]. This possibility is standard to environmental epidemiology studies, and our use of sophisticated, spatially and temporally resolved models to estimate individualized residential pollutant exposures has likely reduced misclassification relative to fixed monitoring studies. The exposure model also did not account for indoor pollutant levels or residential indoor temperatures, and did not estimate the rate at which outdoor pollutants may penetrate indoors, though studies have shown that the contribution of outdoor air pollution to indoor pollutant levels [46, 47] and to monitored personal exposures, including in the Boston area [48], is fairly high.

Our study did not adjust for multiple comparisons. We chose to qualitatively examine the pattern of results instead. As we noted earlier, the confidence intervals associated with most of the observed effect estimates were wide, especially when evaluating PM_2.5_, and only a handful of effect estimates were statistically significant. Our findings are consistent with the greater literature on this subject, which adds plausibility to the pattern of associations we observed. Larger studies with greater statistical power will be needed to confirm and expand on our initial findings.

Ideally, we would have compared results for PM_2.5_ and BC estimated from the same exposure models. However, this was not possible in our study because PM_2.5_ levels were derived from AOD measurements, which do not capture the BC component of PM, and many of the BC measurements were obtained from ethylometers, which measured BC but not overall PM. Thus, we cannot rule out that differences in our findings for BC and fine PM could be due to differing modeling approaches.

The changes in HRV that we observed in this study do not rise to a level of clinical concern, but shifting the distribution of HRV measures may be of public health concern because of changes among ‘borderline’ individuals. Furthermore, PM_2.5_ levels in the Boston area are generally below EPA air quality standards, whereas other areas may have consistently higher pollutant levels which could affect HRV to a greater extent. Finally, part of our goal was to identify mechanisms mediating the association between particulate pollutant exposure and adverse cardiovascular outcomes, and these results, if confirmed, suggest that effects on HRV may be part of that pathway.

Our study population is comprised of elderly men, the vast majority of whom are retired, which is both a limitation and strength. Participants’ estimated residential exposures are likely to reflect the great majority of their total exposure burden, which is a strength in terms of exposure assessment, but results may not be generalizable to other (non-elderly, non-White, female) segments of the population. We examined this association within an elderly cohort because the elderly comprise a large, susceptible sub-population with respect to cardiovascular ailments and health effects of particle exposure. Finally, the sampling rate we used when measuring HRV is still clinically appropriate, but is lower that what is currently preferred for research purposes. This could lead to some non-differential measurement error and a consequent reduction in statistical power and bias towards the null.

Strengths of our study include access to a large, general population cohort with extensive and repeated information regarding pollutant exposures, potential confounders and effect modifiers, and HRV data from multiple study visits. While most air pollution studies examine short-term exposures, longer-term exposures may be of most interest when assessing risk for chronic disease and dysfunction. Hence, an additional strength of our study was the reconstruction of participants’ sub-chronic and long-term BC and PM_2.5_ exposures using comprehensive models. Our use of spatially resolved residential exposure measurements is another important strength, especially for BC, since traffic exposures can vary significantly within a small geographic distance [25].

## Conclusions

In our study of particulate air pollution exposure and HRV, we report a pattern of associations between sub-chronic BC exposure estimates and decreased HF, LF, and SDNN, as well as increased LF:HF ratio. Although our limited statistical power led to wide confidence intervals that often spanned the null, our findings were similar to previous studies of short-term exposure in the NAS [10] and other study populations [14]. Long-term BC exposure was not linked to HRV after adjustment for sub-chronic exposure levels. We report discrepant findings for BC and PM_2.5_, as sub-chronic and long-term PM_2.5_ exposure estimates were associated with an increased LF and LF:HF ratio, but not other changes in HRV (HF or SDNN). HRV may mediate associations between particulate exposures and adverse cardiovascular outcomes, and assessing longer-term exposures is important to understanding effects of air pollution on chronic disease and dysfunction. Future high-powered studies should therefore further evaluate associations between sub-chronic or long-term pollutant exposures and HRV, including with respect to identifying potentially susceptible subpopulations.

## Additional file

Additional file 1: Table S1. Spearman correlation coefficients between selected exposure measurement intervals for BC and PM_2.5_^a^. BC: black carbon; PM_2.5_: particulate matter <2.5 μm in aerodynamic diameter a. Normative Aging Study (2000–2011). (DOC 39 kb)

## Abbreviations

8-OhdG: 8-hydroxydeoxyguanosine
AOD: aerosol optical depth
BC: black carbon
BMI: body mass index
*CAT*: catalase
DBP: diastolic blood pressure
ECG: electrocardiogram
FBG: fasting blood glucose
*GC*: group-specific component
*GCLM*: glutamate-cysteine ligase, modifier subunit
HF: high frequency power
*HMOX1*: heme oxygenase 1
HRV: heart rate variability
LF: low frequency power
MAP: mean arterial blood pressure
NAS: Normative Aging Study
*NQO1*: NAD(P)H dehydrogenase, quinone 1
PM10: particulate matter <10 μm in aerodynamic diameter
PM_2.5_: particulate matter <2.5 μm in aerodynamic diameter
SBP: systolic blood pressure
SD: standard deviation
SDNN: standard deviation of normal-to-normal intervals
SE: standard error
