# Supplementary material for: Exposure to sub-chronic and long-term particulate air pollution and heart rate variability in an elderly cohort: the Normative Aging Study
Source: Environ Health. 2015 Nov 6;14:87. doi: 10.1186/s12940-015-0074-z (PMC4636903; doi:10.1186/s12940-015-0074-z)
Supplement: Additional file 1: Table S1. — Spearman correlation coefficients between selected exposure measurement intervals for BC and PM2.5 a. BC: black carbon; PM2.5: particulate matter <2.5 μm in aerodynamic diameter a. Normative Aging Study (2000–2011). (DOC 39 kb) [file 12940_2015_74_MOESM1_ESM.doc]

Additional file 1: Table S1. Spearman correlation coefficients between selected exposure measurement intervals for BC and PM_2.5_ ^a^

| **Exposure Interval** | 3 days | 14 days | 28 days | 84 days | 1 year |
| --- | --- | --- | --- | --- | --- |
| **BC** | | | | | |
| 3 days | 1 | 0.86 | 0.82 | 0.73 | 0.65 |
| 14 days |  | 1 | 0.97 | 0.87 | 0.74 |
| 28 days |  |  | 1 | 0.92 | 0.76 |
| 84 days |  |  |  | 1 | 0.81 |
| 1 year |  |  |  |  | 1 |
| **PM_2.5_** | | | | | |
| 3 days | 1 | 0.67 | 0.55 | 0.25 | 0.32 |
| 14 days |  | 1 | 0.87 | 0.52 | 0.46 |
| 28 days |  |  | 1 | 0.71 | 0.53 |
| 84 days |  |  |  | 1 | 0.65 |
| 1 year |  |  |  |  | 1 |

BC: black carbon; PM_2.5_: particulate matter <2.5 micrometers in aerodynamic diameter

a. Normative Aging Study (2000-2011)
